# Supplementary material for: Rapidly shifting immunologic landscape and severity of SARS-CoV-2 in the Omicron era in South Africa
Source: Nat Commun. 2023 Jan 16;14:246. doi: 10.1038/s41467-022-35652-0 (PMC9842214; doi:10.1038/s41467-022-35652-0)
Supplement: Supplementary file 1 — Supplementary Information [file 41467_2022_35652_MOESM1_ESM.pdf]

Supplementary Information for

Rapidly shifting immunologic landscape and severity of SARS-CoV-2 in the Omicron era in South Africa

Authors: Kaiyuan Sun[1*], Stefano Tempia[2,3,4], Jackie Kleynhans[2,3], Anne von Gottberg[2,5], Meredith L McMorrow[6], Nicole Wolter[2,5], Jinal N. Bhiman[7,8], Jocelyn Moyes[2,3], Maimuna Carrim[2,5], , Neil A Martinson[9,10], Kathleen Kahn[11], Limakatso Lebina[9], Jacques D. du Toit[11], Thulisa Mkhencele[2], Cécile Viboud[1†], Cheryl Cohen[2,3†*], the PHIRST-C group

Affiliations:
[1]Division of International Epidemiology and Population Studies, Fogarty International Center, National Institutes of Health, Bethesda, Maryland, United States of America.

[2]Centre for Respiratory Diseases and Meningitis, National Institute for Communicable Diseases of the National Health Laboratory Service, Johannesburg, South Africa.

[3]School of Public Health, Faculty of Health Sciences, University of the Witwatersrand, Johannesburg, South Africa.

[4]Influenza Division, Centers for Disease Control and Prevention, Atlanta, Georgia, United States of America.

[5]School of Pathology, Faculty of Health Sciences, University of the Witwatersrand, Johannesburg, South Africa.

[6]COVID-19 Response, Centers for Disease Control and Prevention, Atlanta, Georgia, United States.

[7]Centre for HIV and STIs, National Institute for Communicable Diseases of the National Health Laboratory Service, Johannesburg, South Africa

[8]SAMRC Antibody Immunity Unit, University of the Witwatersrand, Johannesburg, South Africa

[9]Perinatal HIV Research Unit, University of the Witwatersrand, South Africa.

[10]Johns Hopkins University Center for TB Research, Baltimore, Maryland, United States of America.

[11]MRC/Wits Rural Public Health and Health Transitions Research Unit (Agincourt), School of Public Health, Faculty of Health Sciences, University of the Witwatersrand, Johannesburg, South Africa.

*Corresponding author. Email: kaiyuan.sun@nih.gov (KS); cherylc@nicd.ac.za (CC)

† These are co-senior authors of this work.

Table S1: Categorization and infection outcome calibration of the 3rd wave serial serologic patterns (BDs 5, 6, 8, Delta wave). The outcome is a SARS-CoV-2 infection occurring between the mid-points of BD 5 and BD 6, and BD 8. These categories are used to calibrate a serology-based approach to infer infections during a the period of intense cohort follow up period, when rRT-PCR confirmed infections are available (detailed in Methods Section 3.2-3.3).

**Categorization (crude)**			**Categorization (refined#)**			**Infection outcome****
**Category (crude)**	**Criteria**	**Description**	**Subcategory (refined)**	**Criteria**	**ΔJ***	$\gamma = 1.4$
A	$BD_5 < 1$ & $BD_6 < 1$ & $BD_8 < 1$	Seronegative across BD 5, 6, and 8	A	NA	-0.447	Non-infection
B	$BD_5 \geq 1$ & $BD_6 < BD_5$ & $BD_8 < BD_6$	Seropositive at BD5 followed by waning COI from BD 5 to BD 6 and BD 6 to BD 8	B	NA	-0.264	Non-infection
C	$BD_5 \geq 1$ & $BD_6 > BD_5$ & $BD_8 < BD_6$	Seropositive at BD5 followed by boosting COI from BD5 to BD6 and waning COI from BD6 to BD8	C_1	$BD_6/BD_5 > \gamma$	-0.028	Non-infection
			C_0	$BD_6/BD_5 \leq \gamma$	-0.026	Non-infection
D	$BD_5 < 1$ & $BD_6 > 1$ & $BD_8 < BD_6$	Seronegative at BD5 followed by seroconversion at BD6 then waning COI from BD6 to BD8	D	NA	-0.009	Non-infection
E	$BD_5 \geq 1$ & $BD_6 > BD_5$ & $BD_8 > BD_6$	Seropositive at BD5 followed by boosting COI from BD5 to BD6 and another boosting COI from BD6 to BD8	E_2	$BD_8/BD_6 > \gamma$	0.004	Reinfection
			E_1	$BD_8/BD_6 \leq \gamma$ & $BD_6/BD_5 > \gamma$	-0.003	Non-infection
			E_0	$BD_8/BD_6 \leq \gamma$ & $BD_6/BD_5 \leq \gamma$	-0.007	Non-infection
F	$BD_5 < 1$ & $BD_6 \geq 1$ & $BD_8 > BD_6$	Seronegative at BD5 followed by seroconversion at BD6 and boosting COI from BD6 to BD8	F_1	$BD_8/BD_6 > \gamma$	0.017	Reinfection†
			F_0	$BD_8/BD_6 \leq \gamma$	-0.008	Non-infection
G	$BD_5 \geq 1$ & $BD_6 < BD_5$ & $BD_8 > BD_6$	Seropositive at BD5 followed by waning COI from BD5 to BD6 and boosting COI from BD6 to BD8	G_1	$BD_8/BD_6 > \gamma$	0.061	Reinfection
			G_0	$BD_8/BD_6 \leq \gamma$	-0.029	Non-infection
H	$BD_5 < 1$ & $BD_6 < 1$ & $BD_8 \geq 1$	Seronegative at BD5 and BD6 followed by seroconversion at BD8	H	NA	0.738	Primary infection or reinfection‡

*ΔJ: the net contribution of the Youden's J statistics of a particular serologic category, if the category were considered as a marker for infection (detailed in Section 3.3)

**Outcome of whether a specific serologic category is considered as a marker of infection between May 1, 2021 (mid-point of BD 5 and BD 6), and BD 8.

#Refined categorization differentiating strong vs weak boosting signal to account for potential measurement noise.

†In rare occasions (Figure S4 C), this could also be primary infection occurred right before BD 6, with COI yet to reach high level at BD 6 and COI at BD 8 still significantly higher than that of BD 6 even after waning.

‡This could be primary infection seroconverted between BD 6 and BD 8 or reinfection between BD 6 and BD 8 with prior infection sero-reverted at BD 5 and BD 6 (Figure S4 C-D).

Table S2: Categorization of the 4th wave serial serologic patterns (BDs 8, 9, 10, Omicron wave). The determination of infections outcomes (last column) for each subcategory of sequential serologic patterns A-H are based on the calibration detailed in Table S1. This approach captures infections and re-infections that occurred between the mid-point of BD 8 and BD 9, and BD 10, during the Omicron BA1/2 wave.

Wave	Categorization (crude)			Categorization (refined#)		Infection outcome*
	**Category (crude)**	**Criteria**	**Description**	**Subcategory (refined)**	**Criteria**	$\gamma = 1.4$
**4th epidemic wave in South Africa**	A	$BD_8 < 1$ & $BD_9 < 1$ & $BD_{10} < 1$	Seronegative across BD 8, 9, and 10	A	NA	Non-infection
	B	$BD_8 \geq 1$ & $BD_9 < BD_8$ & $BD_{10} < BD_9$	Seropositive at BD8 followed by waning COI from BD 8 to BD 9 and BD 9 to BD 10	B	NA	Non-infection
	C	$BD_8 \geq 1$ & $BD_9 > BD_8$ & $BD_{10} < BD_9$	Seropositive at BD8 followed by boosting COI from BD8 to BD9 and waning COI from BD9 to BD10	C_1	$BD_9/BD_8 > \gamma$	Non-infection
				C_0	$BD_9/BD_8 \leq \gamma$	Non-infection
	D	$BD_8 < 1$ & $BD_9 > 1$ & $BD_{10} < BD_9$	Seronegative at BD8 followed by seroconversion at BD9 then waning COI from BD9 to BD10	D	NA	Non-infection
	E	$BD_8 \geq 1$ & $BD_9 > BD_8$ & $BD_{10} > BD_9$	Seropositive at BD8 followed by boosting COI from BD8 to BD9 and another boosting COI from BD9 to BD10	E_2	$BD_{10}/BD_9 > \gamma$	Reinfection
				E_1	$BD_{10}/BD_9 \leq \gamma$ & $BD_9/BD_8 > \gamma$	Non-infection
				E_0	$BD_{10}/BD_9 \leq \gamma$ & $BD_9/BD_8 \leq \gamma$	Non-infection
	F	$BD_8 < 1$ & $BD_9 \geq 1$ & $BD_{10} > BD_9$	Seronegative at BD8 followed by seroconversion at BD9 and boosting COI from BD9 to BD10	F_1	$BD_{10}/BD_9 > \gamma$	Reinfection†
				F_0	$BD_{10}/BD_9 \leq \gamma$	Non-infection
	G	$BD_8 \geq 1$ & $BD_9 < BD_8$ & $BD_{10} > BD_9$	Seropositive at BD8 followed by waning COI from BD8 to BD9 and boosting COI from BD9 to BD10	G_1	$BD_{10}/BD_9 > \gamma$	Reinfection
				G_0	$BD_{10}/BD_9 \leq \gamma$	Non-infection
	H	$BD_8 < 1$ & $BD_9 < 1$ & $BD_{10} \geq 1$	Seronegative at BD8 and BD9 followed by seroconversion at BD10	H	NA	Primary infection or infection‡

*Outcome of whether a specific serologic category is considered as a marker of infection between mid-point (of BD 8 and BD 9), and BD 10.
#Refined categorization differentiating strong vs weak boosting signal to account for potential measurement noise.
†In rare occasions, this could also be primary infection occurred right before BD 9, with COI yet to reach high level at BD 9 and COI at BD 10 still significantly higher than that of BD 9 even after waning.
‡This could be primary infection seroconverted between BD 9 and BD 10 or reinfection between BD 9 and BD 10 with prior infection sero-reverted at BD 8 and BD 9.

Table S3: Model selections on the covariates of the chain-binomial models.

Model	0	1	2	3	4	5	6	7	8	9	10
-Log(likelihood)	1146	1126	1095	1094	1078	1078	1055	1054	1044	991	990
Akaike Information Criterion (AIC)	2300	2262	2202	2202	2174	2178	2136	2138	2021	2016	2016
ΔAIC	0	-38	-98	-98	-126	-122	-164	-162	-180	-284	-284
**Parameters**											
Baseline household transmission risk (rural)	*	*	*	*	*	*	*	*	*	*	*
Baseline household transmission risk (urban)	*	*	*	*	*	*	*	*	*	*	*
Baseline community infection risk (rural)	*	*	*	*	*	*	*	*	*	*	*
Baseline community infection risk (urban)	*	*	*	*	*	*	*	*	*	*	*
Variant type		*	*	*	*	*	*	*	*	*	*
Household size			*	*	*	*	*	*	*	*	*
If living with HIV				*	*	*	*	*	*	*	*
Age (rural household risk)					*	*	*	*	*	*	*
Age (urban household risk)					*	*	*	*	*	*	*
Sex (rural household risk)						*	*	*	*	*	*
Sex (urban household risk)						*	*	*	*	*	*
Age (rural community risk)							*	*	*	*	*
Age (urban community risk)							*	*	*	*	*
Sex (rural community risk)								*	*	*	*
Sex (urban community risk)								*	*	*	*
If reinfection									*	*	*
Prior exposure										*	*
Time since last infection											*

* Indicates covariates included in the model.

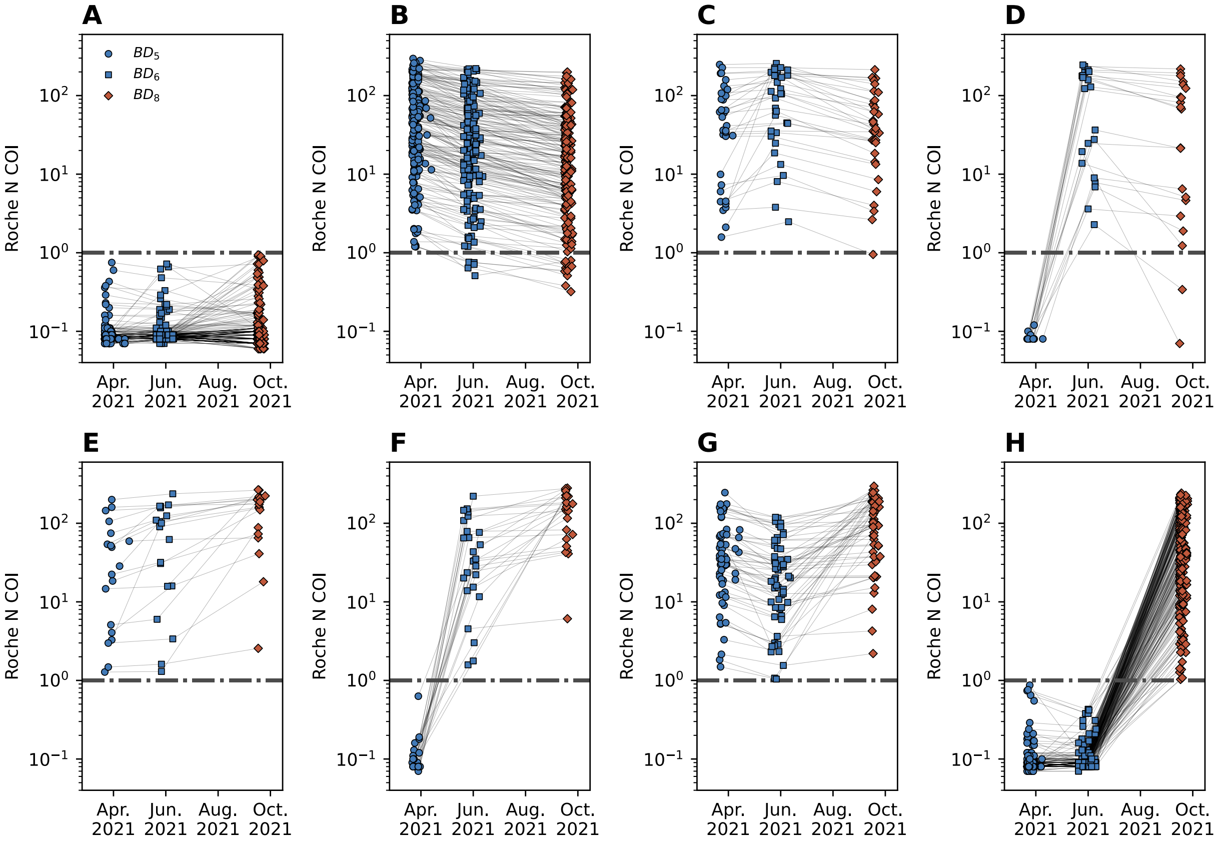

Figure S1: Panel A-H, The serologic trajectory for Roche anti-N COIs at BD 5, BD 6, and BD 8 for each of the crude serologic categories A, B, C, D, E, F, G, and H, respectively. The timing of the BDs bounds SARS-CoV-2 infections during South Africa's 3rd epidemic wave (Figure 1). The horizontal dashed line indicates the Roche anti-N COI reactivity threshold for seropositivity.

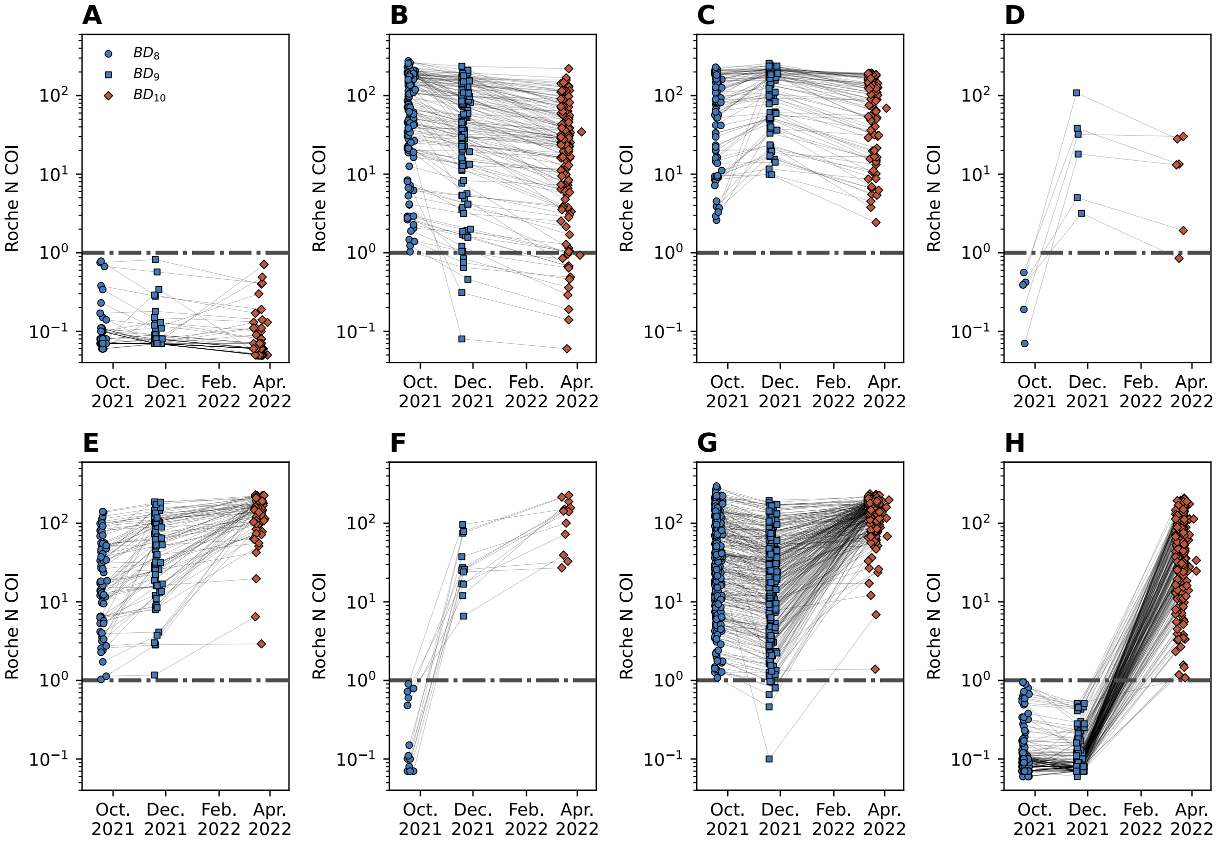

Figure S2: Panel A-H, The serologic trajectory for Roche anti-N COIs at BD 8, BD 9, and BD 10 for each of the crude serologic categories A, B, C, D, E, F, G, and H, respectively. The timing of the BDs bounds SARS-CoV-2 infections during South Africa's 4th epidemic wave (Figure 1). The horizontal dashed line indicates the Roche anti-N COI reactivity threshold for seropositivity.

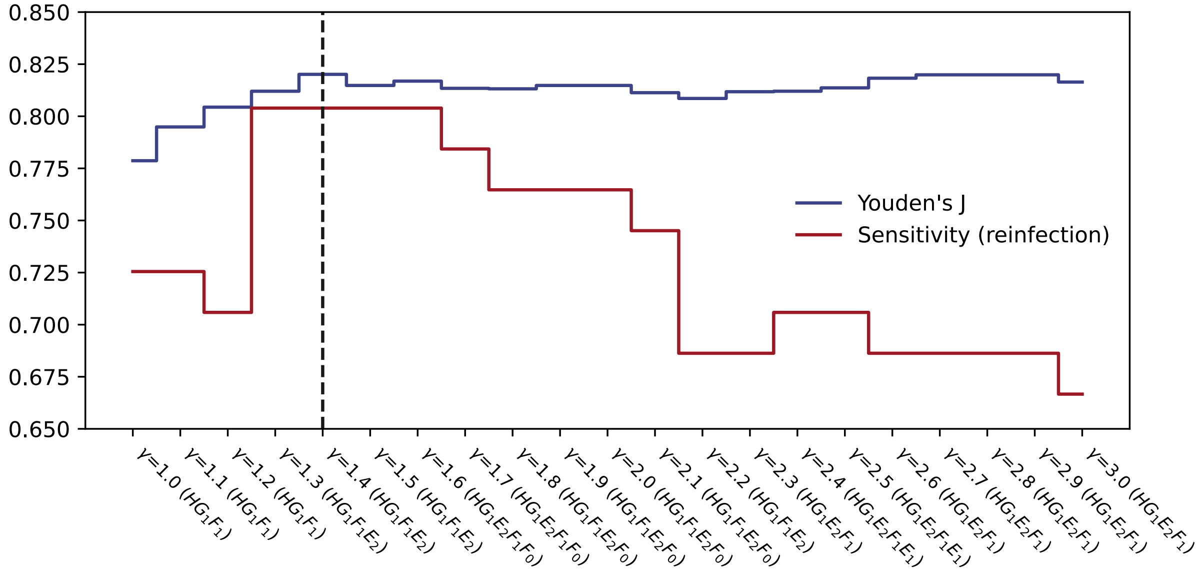

Figure S3: The optimized serial serologic patterns to categorize SARS-CoV-2 infections by Youden's J statistics and the corresponding sensitivity for reinfections. We plot the Youden's J statistics (blue line) and the sensitivity to detect Delta reinfection (red line) based on function of the boosting threshold γ (in the x axis's tick labels, we listed the corresponding refined serologic categories (Table S1) selected as a marker of past SARS-CoV-2 infections).

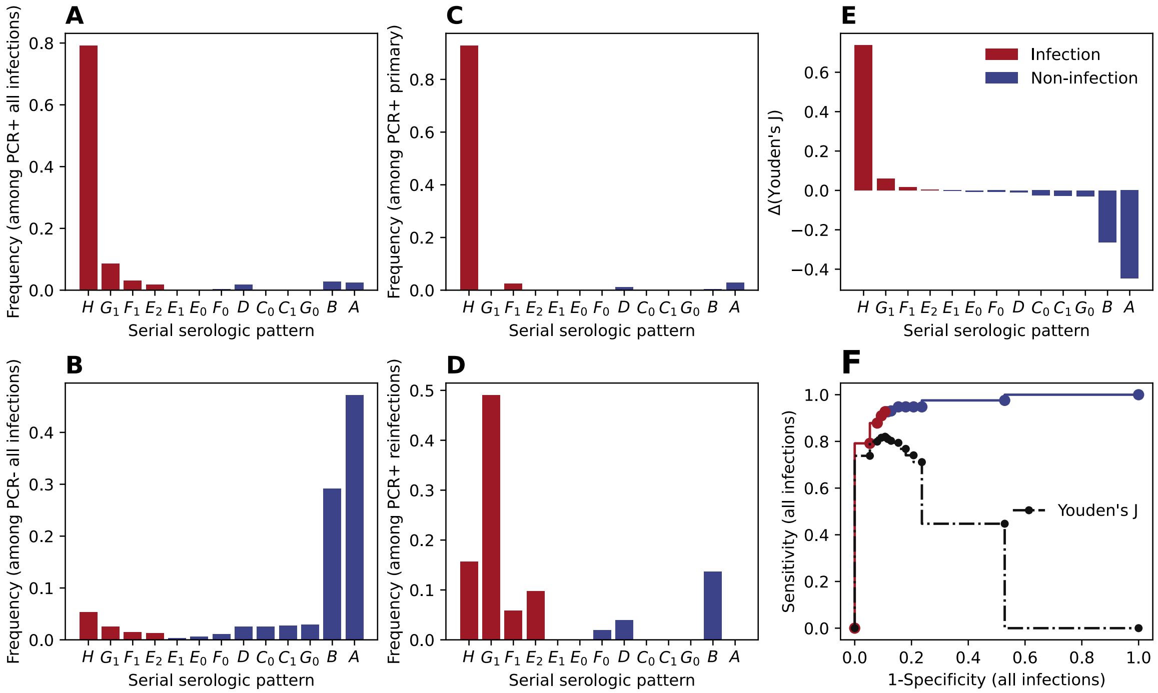

Figure S4: For $\gamma = 1.4$, The optimized serial serologic patterns to categorize SARS-CoV-2 infections by Youden's J statistics (blue line) and the corresponding sensitivity for reinfections (red line). Panel A: the distribution of serologic patterns among all rRT-PCR confirmed infection (PCR+). B: the distribution of serologic patterns among all rRT-PCR confirmed non-infection (PCR-). C: same as A but among primary infections, D, same as A but among reinfections infections. E, net-contribution of Youden's J statistic for each of the serial serologic patterns. F, the receiver operating characteristic curve (ROC) by incorporating serial serologic patterns one by one in the order of the net contribution to Youden's J statistics (panel E); the dashed line is the overall Youden's J statistics. Across all panels, reds are serial serologic patterns selected as markers for SARS-CoV-2 infection (with net-positive contribution to Youden's J), while blues are serial serologic patterns selected as marks for SARS-CoV-2 non-infection.

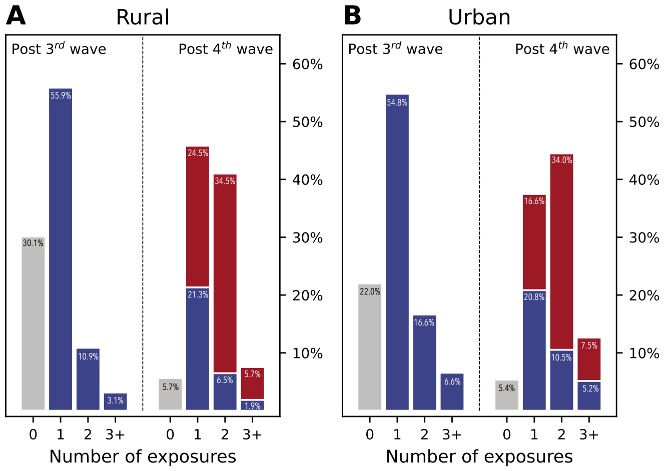

Figure S5: A: The distribution of the rural site's cohort population by the number of SARS-CoV-2 exposures experienced after the 3rd epidemic wave (left) or after the 4th epidemic wave (right). Each infection/vaccine dose is counted as 1 exposure. Gray bars represent naïve individuals; blue bars represent individuals who have been infected by pre-Omicron variants or received SARS-CoV-2 vaccinations; red bars represent individuals who have been infected by Omicron. B: Same as A but for the urban site.

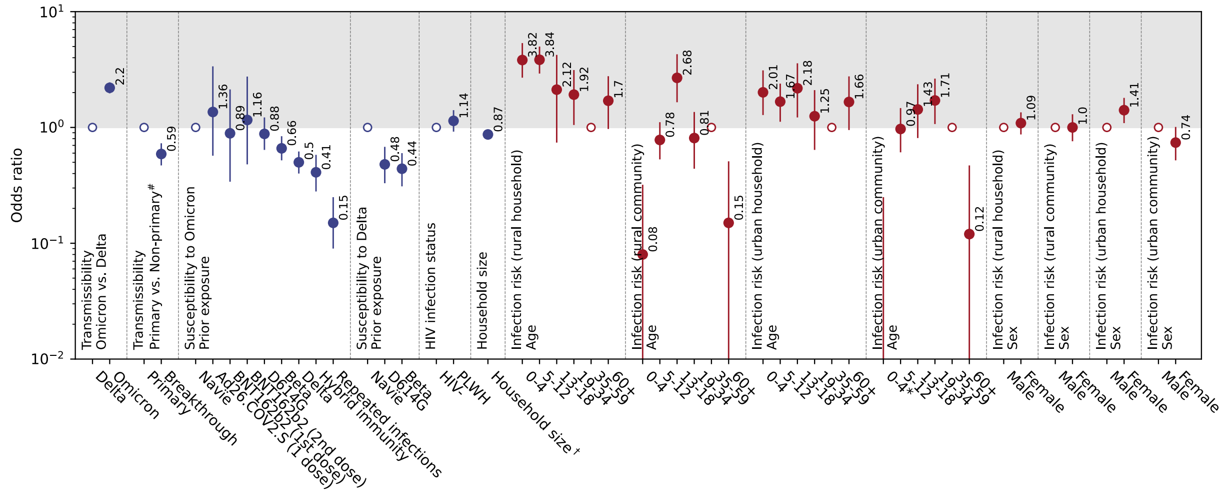

Figure S6: Sensitivity analysis of risk factors associated with SARS-CoV-2 Omicron BA.1/2 and Delta infection. Odds ratios (adjusted after controlling for other risk factors, see Methods Section 4 for details) were estimated by a chain binomial model fitted to the infection outcome of n=905 participants. Omicron BA.1/2 infections were inferred by the serologic approach while Delta infections were either inferred by serologic approach or confirmed by rRT-PCR. Empty circles are reference classes. Solid dots and lines represent maximum likelihood estimate and 95% confidence intervals.

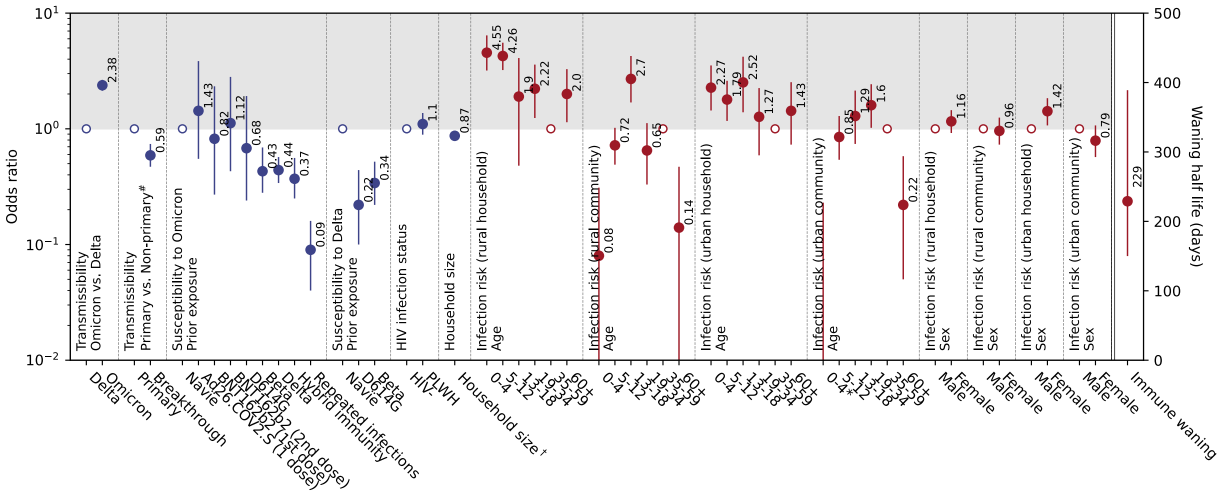

Figure S7: Risk factors associated with SARS-CoV-2 Omicron BA.1/2 and Delta infection, adjusted for waning of immunity. Odds ratios and waning half-life (adjusted after controlling for other risk factors, see Methods Section 4 for details) were estimated by a chain binomial model fitted to the infection outcome of n=905 participants. Omicron BA.1/2 and Delta infections were inferred by the serologic approach. Empty circles are reference classes. Solid dots and lines represent maximum likelihood estimate and 95% confidence intervals.

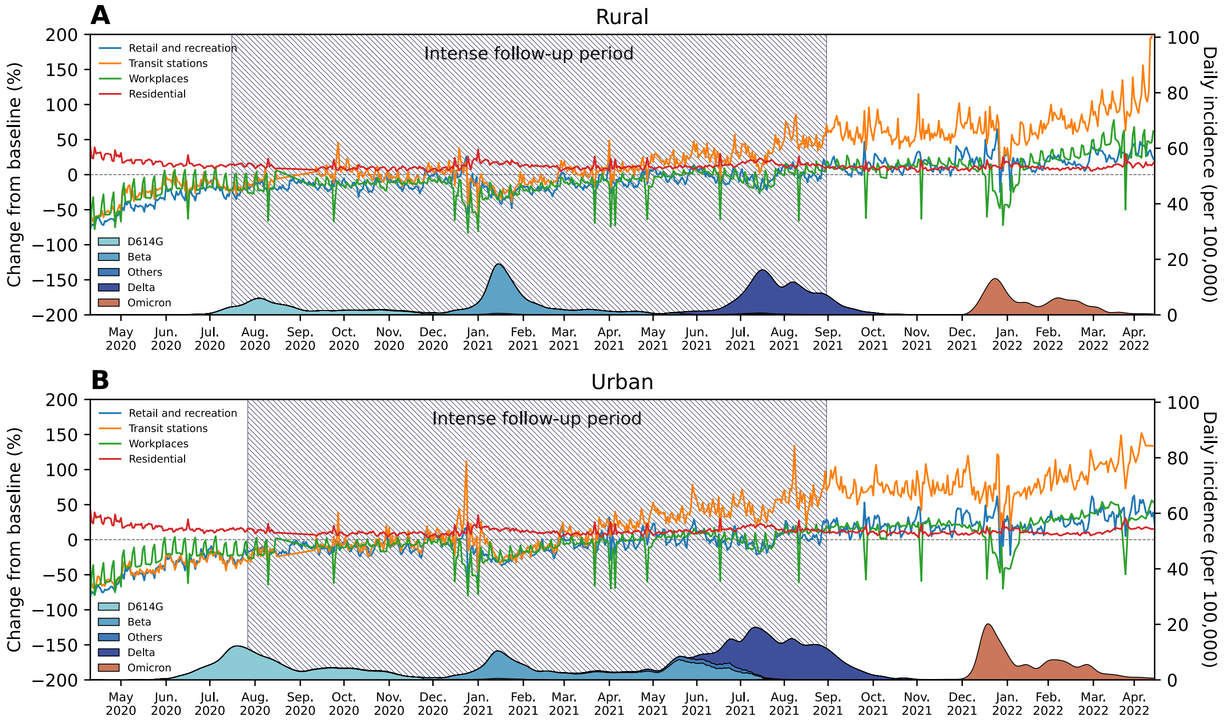

Figure S8: Community mobility trends in South Africa, based on Google's Community Mobility Reports (*1*)**.** (A) Lines of different colors represent the percentage change of Google's mobility index in different setting (*1*) in Mpumalanga Province (rural site). The shaded curve at the bottom represents the daily incidence of SARS-CoV-2 cases in routine surveillance data collected by the Ehlanzeni District, Mpumalanga Province. Colors of the shaded curve represent different variant types. Here, blood draw (BD) 10 was collected at the end of the first Omicron wave. Since in South Africa, Omicron BA.4 and BA.5 only started to rise at April, 2022 (*2*), we assume the Omicron wave prior to BD 10 were BA.1 and BA.2 subvariants. The hatched area represents the period of intense follow-up of the PHIRST-C cohort, when nasal swabs were collected and tested on rRT-PCR at twice-a-week frequency. (B) Same as (A) but for the North West Province (Urban).

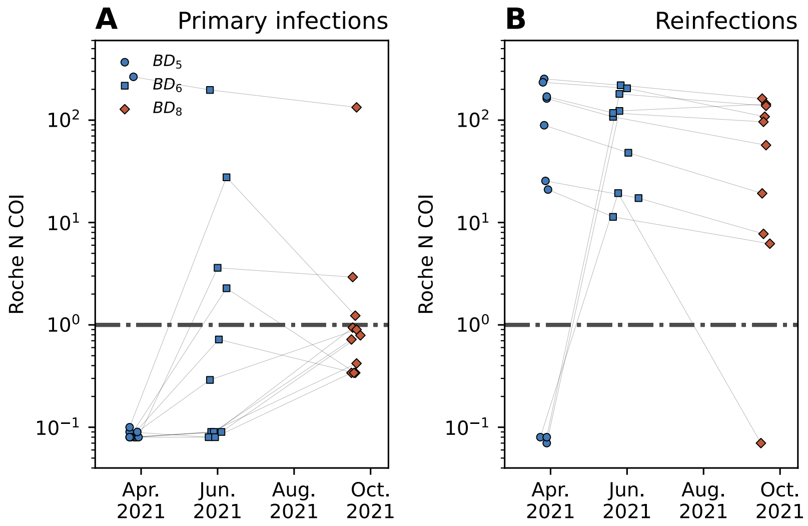

Figure S9: The serologic trajectory for Roche anti-N COIs at BD 5, BD 6, and BD 8 for the 11 rRT-PCR confirmed Delta primary infections (A) and 10 Delta reinfections (B) missed by the serologic approach. The horizontal dashed lines indicate the Roche anti-N COI reactivity threshold for seropositivity.

Supplementary references

1. Google, COVID-19 Community Mobility Reports, (available at https://www.google.com/covid19/mobility/).

2. H. Tegally, M. Moir, J. Everatt, M. Giovanetti, C. Scheepers, E. Wilkinson, K. Subramoney, Z. Makatini, S. Moyo, D. G. Amoako, C. Baxter, C. L. Althaus, U. J. Anyaneji, D. Kekana, R. Viana, J. Giandhari, R. J. Lessells, T. Maponga, D. Maruapula, W. Choga, M. Matshaba, M. B. Mbulawa, N. Msomi, NGS-SA consortium, Y. Naidoo, S. Pillay, T. J. Sanko, J. E. San, L. Scott, L. Singh, N. A. Magini, P. Smith-Lawrence, W. Stevens, G. Dor, D. Tshiabuila, N. Wolter, W. Preiser, F. K. Treurnicht, M. Venter, G. Chiloane, C. McIntyre, A. O'Toole, C. Ruis, T. P. Peacock, C. Roemer, S. L. K. Pond, C. Williamson, O. G. Pybus, J. N. Bhiman, A. Glass, D. P. Martin, B. Jackson, A. Rambaut, O. Laguda-Akingba, S. Gaseitsiwe, A. von Gottberg, T. de Oliveira, Emergence of SARS-CoV-2 Omicron lineages BA.4 and BA.5 in South Africa. *Nat. Med.* (2022), doi:10.1038/s41591-022-01911-2.
