# Supplementary material for: Rapidly shifting immunologic landscape and severity of SARS-CoV-2 in the Omicron era in South Africa
Source: Nat Commun. 2023 Jan 16;14:246. doi: 10.1038/s41467-022-35652-0 (PMC9842214; doi:10.1038/s41467-022-35652-0)
Supplement: Supplementary file 2 — Reporting Summary [file 41467_2022_35652_MOESM2_ESM.pdf]

nature portfolio

Corresponding author(s): Kaiyuan Sun
Cheryl Cohen

Last updated by author(s): Dec 3, 2022

Reporting Summary

Nature Portfolio wishes to improve the reproducibility of the work that we publish. This form provides structure for consistency and transparency in reporting. For further information on Nature Portfolio policies, see our Editorial Policies and the Editorial Policy Checklist.

Statistics

For all statistical analyses, confirm that the following items are present in the figure legend, table legend, main text, or Methods section.

n/a	Confirmed	
☐	☒	The exact sample size (n) for each experimental group/condition, given as a discrete number and unit of measurement
☐	☒	A statement on whether measurements were taken from distinct samples or whether the same sample was measured repeatedly
☒	☐	The statistical test(s) used AND whether they are one- or two-sided<br>*Only common tests should be described solely by name; describe more complex techniques in the Methods section.*
☐	☒	A description of all covariates tested
☒	☐	A description of any assumptions or corrections, such as tests of normality and adjustment for multiple comparisons
☒	☐	A full description of the statistical parameters including central tendency (e.g. means) or other basic estimates (e.g. regression coefficient) AND variation (e.g. standard deviation) or associated estimates of uncertainty (e.g. confidence intervals)
☒	☐	For null hypothesis testing, the test statistic (e.g. F, t, r) with confidence intervals, effect sizes, degrees of freedom and P value noted<br>*Give P values as exact values whenever suitable.*
☒	☐	For Bayesian analysis, information on the choice of priors and Markov chain Monte Carlo settings
☒	☐	For hierarchical and complex designs, identification of the appropriate level for tests and full reporting of outcomes
☒	☐	Estimates of effect sizes (e.g. Cohen's d, Pearson's r), indicating how they were calculated

Our web collection on statistics for biologists contains articles on many of the points above.

Software and code

Policy information about availability of computer code

Data collection	Data were entered during visits on tablet computers with use of the Research Electronic Data Capture application (REDCap).
Data analysis	Software packages used: python version 3.8.11; scipy version 1.7.1. Code to reproduce the figures, and the chain-binomial transmission model are available at https://doi.org/10.5281/zenodo.7260083

For manuscripts utilizing custom algorithms or software that are central to the research but not yet described in published literature, software must be made available to editors and reviewers. We strongly encourage code deposition in a community repository (e.g. GitHub). See the Nature Portfolio guidelines for submitting code & software for further information.

Data

Policy information about availability of data

All manuscripts must include a data availability statement. This statement should provide the following information, where applicable:

- Accession codes, unique identifiers, or web links for publicly available datasets
- A description of any restrictions on data availability
- For clinical datasets or third party data, please ensure that the statement adheres to our policy

All data associated with this study are present in the paper or the Supplementary Information. To access individual participant data and a data dictionary defining each field in the dataset, please submit a proposal to CC. These data can be made available through a data access agreement or material transfer agreement. Aggregate data to reproduce the figures, and the chain-binomial transmission model are available at https://doi.org/10.5281/zenodo.7260083.

Human research participants

Policy information about studies involving human research participants and Sex and Gender in Research.

Reporting on sex and gender	Information on sex were collected based on self-reporting.
Population characteristics	Participant characteristics are summarized in Table 1 of the manuscript.
Recruitment	Households were randomly selected, from the HDSS database for the rural site and with use of GPS coordinates for the urban site (appendix p 1 of https://doi.org/10.5281/zenodo.7260083). Households with more than two members and where at least 75% of members consented to participate were eligible. In brief, we first approached households previously enrolled in PHIRST, and then prospectively approached new potentially eligible households using the site-specific sampling frame used for PHIRST until the required number of households were enrolled. For households that failed to recruit all members the infection status of household members that were not recruited remained unknown, which could underestimate the force-of-infection within the household setting for the transmission model. Exclusion of household with <2 members could have biased estimates of attack rates.
Ethics oversight	The PHIRST-C protocol was approved by the University of Witwatersrand Human Research Ethics Committee (Reference 150808) and the U.S. Centers for Disease Control and Prevention's Institutional Review Board relied on the local review (#6840). Informed consent was obtained from all adult participants (aged ≥18 years), assent from children aged 7 to 17 years, and consent from a parent or guardian for children younger than 18 years before data collection. Participants receive grocery store vouchers of ZAR50 (USD 3) per visit to compensate for time required for specimen collection and interview.

Note that full information on the approval of the study protocol must also be provided in the manuscript.

Field-specific reporting

Please select the one below that is the best fit for your research. If you are not sure, read the appropriate sections before making your selection.

☒ Life sciences ☐ Behavioural & social sciences ☐ Ecological, evolutionary & environmental sciences

For a reference copy of the document with all sections, see nature.com/documents/nr-reporting-summary-flat.pdf

Life sciences study design

All studies must disclose on these points even when the disclosure is negative.

Sample size	NA (The original sample size calculation for the primary endpoints of the PHIRST-C cohort is described at https://doi.org/10.1016/S1473-3099(22)00069-X, this study represents analysis of the cohort data that was not planned during the original study design)
Data exclusions	The analysis was performed on a subset (905) of the total 1,200 cohort participants, who had complete serum specimens collections during the relevant Delta and Omicron periods.
Replication	NA (This is a prospective household observational study during the COVID-19 pandemic, circulating variants and population immunity have fundamentally changed throughout the study thus it is impossible to replicate the real-world epidemiological situations in a similar population)
Randomization	NA (covariates were controlled by fitting a multi-variable chain-binomial model
Blinding	NA (This is a prospective household observational study, no blinding performed)

Reporting for specific materials, systems and methods

We require information from authors about some types of materials, experimental systems and methods used in many studies. Here, indicate whether each material, system or method listed is relevant to your study. If you are not sure if a list item applies to your research, read the appropriate section before selecting a response.

Materials & experimental systems

n/a	Involved in the study
☒	☐ Antibodies
☒	☐ Eukaryotic cell lines
☒	☐ Palaeontology and archaeology
☒	☐ Animals and other organisms
☐	☒ Clinical data
☒	☐ Dual use research of concern

Methods

n/a	Involved in the study
☒	☐ ChIP-seq
☒	☐ Flow cytometry
☒	☐ MRI-based neuroimaging

Clinical data

Policy information about clinical studies

All manuscripts should comply with the ICMJE guidelines for publication of clinical research and a completed CONSORT checklist must be included with all submissions.

Clinical trial registration

NCT02519803; NCT05277298

Study protocol

https://clinicaltrials.gov/ct2/show/NCT02519803; https://clinicaltrials.gov/ct2/show/NCT05277298

Data collection

The study aims to characterize the community burden (including the clinical features) and transmissibility of SARS-CoV-2 within the context of a functional antibody response. In addition,the study will assess the effect of the interaction of SARS-CoV-2 with influenza virus and RSV on disease severity and transmission dynamics. A household-level prospective cohort study will be conducted in one rural and one urban community located in Mpumalanga Province and North West Province, respectively. The study will be conducted for 12 months of intensive follow up (July 2020 to August 2021) with a post-intensive follow-up continuing for a further 16 months (until December 2022). Two hundred households; 1,000 study participants of all ages; will be randomly selected from a list of 327 hoseholds that participated and successfully completed a 10-months follow-up period in a study similar to that currently proposed, but directed at community burden and transmission dynamics of influenza, respiratory syncytial virus and other respiratory pathogens. Each household and household member will be enumerated and the HIV infection status and the level of immunosuppression of HIV-infected individuals will be assessed. Each household member will be followed twice per week during the intense follow-up period (12 months) of the study. During this period upper respiratory tract samples will be collected irrespective of presence of symptoms and data on key symptoms, healthcare seeking, hospitalization and death will be captured at each follow up visit. Respiratory samples will be tested by reverse transcriptase real-time polymerase chain reaction (rRT-PCR) for SARS-CoV-2, influenza and RSV, and selected samples will be cultured and sequenced. An infection risk questionnaire will be administered to all study participants at enrollment and every month thereafter. Sera will be collected at enrollment and every 2 months during the 12-month intense follow-up period from all participants. In addition, sera will be collected every 2 months for a further 6 months following the 12-month intense follow-up period from study participants that tested positive for SARS-CoV-2 by rRT-PCR on respiratory specimens at 14, 16 and 18 months and from all study participants at 18 months. Sera will be tested for the presence of SARS-CoV-2, influenza and RSV antibodies. Wearable proximity sensors will be deployed for 8-12 days in each household over the 6-month intense follow-up period.

Outcomes

1.Describe the symptomatic fraction of SARS-CoV-2 infections among individuals in household cohort in an rural and urban setting, South Africa 2020/2021. [Time Frame: 14 months]
The proportion of SARS-CoV2 infection that are symptomatic. This will be stratified by age, underlying conditions, HIV infection.

2.Describe the household secondary infection risk of SARS-CoV-2 infection among individuals in a household cohort in an rural and urban setting, South Africa 2020/2021. [Time Frame: 14 months]
The number of secondary cases in a household divided by the total number of susceptible gives the secondary attack risk during the 14months of follow-up. This will be explored by underlying conditions, age and HIV status

3.Describe the serial interval for SARS-CoV-2 in households over 14 months of follow-up in an urban and rural setting, South Africa 2020/2021 [Time Frame: 14 months]
Calculate the time between successive cases in a each household as documented by onset of symptoms and/or PCR positive test.

4.Describe the duration of shedding of SARS-CoV-2 in a household cohort in an rural and urban setting, South Africa 2020/2021. [Time Frame: 14 month]
The duration of PCR positive SARS-CoV-2 infection in individual over 14 months of follow-up, including describing the interval by age, HIV status and underlying conditions.

5.Describe the incidence of infection by PCR and serology in a household cohort in a rural and urban setting, South Africa 2020/21 [Time Frame: 14 months]
The number of new cases of symptomatic illness recorded by symptoms reported cohort over the 14 months of follow-up. Including estimating a person time to follow-up incident rate

6.Describe the incidence illness by PCR and/or serology in a household cohort in a rural and urban setting, South Africa 2020/21 [Time Frame: 14 months]
Describe the incidence of infection by PCR and serology in a household cohort over 14 months of follow-up, in a rural and urban setting, South Africa 2020/21
